# Pressure-Insensitive Epidermal Thickness of Fingertip Skin for Optical Image Encryption

**DOI:** 10.3390/s24072128

**Published:** 2024-03-26

**Authors:** Wangbiao Li, Bo Zhang, Xiaoman Zhang, Bin Liu, Hui Li, Shulian Wu, Zhifang Li

**Affiliations:** The Key Laboratory of Optoelectronic Science and Technology for Medicine of Ministry of Education, Fujian Provincial Key Laboratory of Photonics Technology, Fujian Provincial Engineering Technology Research Center for Photoelectric Sensing Application, College of Photonic and Electronic Engineering, Fujian Normal University, Fuzhou 350117, China; lwb@fjnu.edu.cn (W.L.);

**Keywords:** epidermal thickness, cross-sectional OCT image, convolutional neural networks, maximum intensity projection

## Abstract

In this study, an internal fingerprint-guided epidermal thickness of fingertip skin is proposed for optical image encryption based on optical coherence tomography (OCT) combined with U-Net architecture of a convolutional neural network (CNN). The epidermal thickness of fingertip skin is calculated by the distance between the upper and lower boundaries of the epidermal layer in cross-sectional optical coherence tomography (OCT) images, which is segmented using CNN, and the internal fingerprint at the epidermis–dermis junction (DEJ) is extracted based on the maximum intensity projection (MIP) algorithm. The experimental results indicate that the internal fingerprint-guided epidermal thickness is insensitive to pressure due to normal correlation coefficients and the encryption process between epidermal thickness maps of fingertip skin under different pressures. In addition, the result of the numerical simulation demonstrates the feasibility and security of the encryption scheme by structural similarity index matrix (SSIM) analysis between the original image and the recovered image with the correct and error keys decryption, respectively. The robustness is analyzed based on the SSIM value in three aspects: different pressures, noise attacks, and data loss. Key randomness is valid by the gray histograms, and the average correlation coefficients of adjacent pixelated values in three directions and the average entropy were calculated. This study suggests that the epidermal thickness of fingertip skin could be seen as important biometric information for information encryption.

## 1. Introduction

Information in modern society has been closely connected with the Internet. People pay more and more attention to encryption for information protection with the rapid development of information technology [1,2,3,4]. At present, most information security technologies are based on the mathematical theory of cryptography [5,6,7]. However, these digital-based technologies have some limitations, including key generation, distribution, and storage, and malicious attacks still exist.

As a branch of information security research, non-traditional cryptographic theory based on optical principles and technology has played a very important role. It has become an important current research issue [8,9,10,11]. Optical image encryption technology came into being with low costs, high speed, and high parallelism. It is known that light has multiple properties, such as wavelength, amplitude, phase, and polarization state [12,13]. So light is a multi-dimensional information carrier that provides a variety of encryption options and great freedom for the key system of optical image encryption. In 1995, Refregier et al. [14] proposed double-random phase encoding technology. Subsequently, optical image encryption has obtained wide attention, and a large number of optical encryption techniques based on the double-random phase template have been proposed. For example, Situ et al. [15] applied double-random phase encoding technology to the Fresnel domain. Alfalouand et al. [16] used double-random phase encoding technology to realize the multiplexing of image encryption. Although the double-random phase encryption method has achieved some success, it is vulnerable to attack because the double-random phase is a symmetric encryption system, and its linearity reduces the security of the system.

To solve this problem, Qin and Peng [17] proposed an asymmetric encryption system based on the phase-truncated Fourier transform (PTFT). But this asymmetric cryptosystem (ACS) could not be considered a real ACS since the keys should be independent of the plaintext [18]. Vilardy et al. [19] presented a new experimental system for optical encryption using a nonlinear joint transform correlator (JTC) to implement the optical security technique of double-random phase encoding (DRPE). Guo et al. developed a Stokes cell hologram optical encryption scheme by combining visual cryptography with a cell surface-assisted pattern mask. An asymmetric encryption scheme based on Stokes vector rotation transformation is proposed to solve the inherent problem of sharing keys in symmetric encryption [20]. However, the encryption and decryption times have increased.

Biometric features such as fingerprints, iris, faces, and voices are unique to each person and can be used as keys. Thus, information encryption using human biometrics can improve the security of optical image encryption systems because the biometric key is independent of plaintext. In 1994, Tomko et al. applied biometrics to the public key encryption system and proposed the concept of biometric encryption for the first time [21]. The idea is to generate biometric templates by Fourier transform on biometric keys so that attackers cannot directly obtain the original biometric information. From then on, the human fingerprint was widely applied to cryptosystems [22,23,24]. However, surface fingerprints are easily destroyed and imitated. Surface fingerprints have been easily abused, resulting in reduced security. In addition, other existing technologies, such as faces, can be collected non-invasively without permission in daily life, which results in unsafety. Therefore, it is urgent to develop a new key with high security that is not easy to attack.

It has been reported that the internal fingerprint is located at the papillary junction within 220–550 μm of the surface and has the same topography as the surface fingerprint [25]. Optical coherence tomography (OCT) is a noninvasive imaging technique for internal fingerprint acquisition and reconstruction under the finger surface [26,27,28,29,30,31,32]. In our previous work, the 3D fingertip skin based on OCT can be extracted from the epidermal thickness [33], sweat ducts [34], and the internal fingerprint [35]. Therefore, the characteristics of 3D fingertip skin based on OCT have the potential for encryption with high security.

In this study, the internal fingerprint-guided epidermal thickness of fingertip skin based on OCT was proposed as a biometric key for optical image encryption. The algorithm employed in this study combines OCT with the U-Net architecture of a convolutional neural network (CNN) to extract internal fingerprint-guided epidermal thickness. The selection of this approach is motivated by the inherent advantages of U-Net, which include its effectiveness in producing accurate segmentation results and its relatively low demand for training data. Firstly, the influence of pressure on epidermal thickness is investigated since there was a different pressure on the glass when fingertip skin was imaged by OCT at different times. Then the performance of encryption is analyzed by simulating the whole encryption and decryption process [36]. Finally, grayscale histogram, adjacent pixel correlation, and image entropy were used to evaluate the imperceptibility level of the original and fake keys.

## 2. Methods

### 2.1. OCT System and Sample

**Figure 1:**
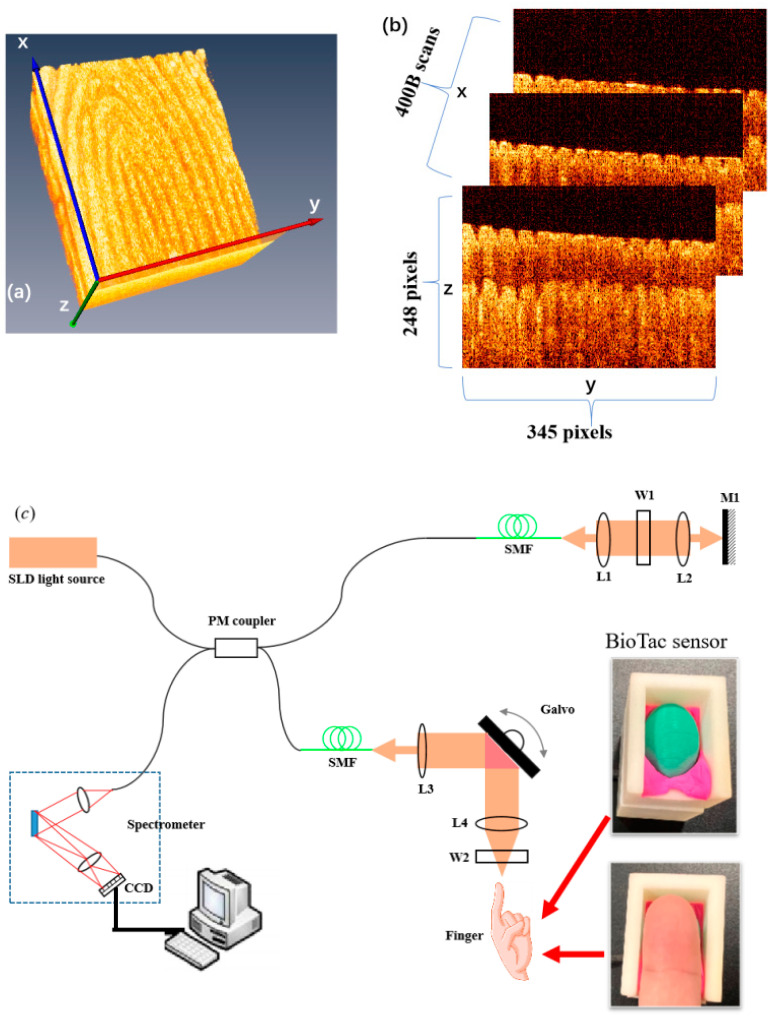
(**a**) Typical 3D OCT image of fingertip skin; (**b**) cross-sectional OCT image of fingertip skin; and (**c**) OCT system combined with BioTac sensor.

A sketch of our spectral domain optical coherence tomography (SD-OCT) system is shown in Figure 1c. The light source is a 12 mw super luminescent diode (SLD) with a FWHM bandwidth of 85 nm centered at 1310 nm (S5FC1021P, Thorlabs, Newton, NC, USA). The light is delivered into a coupler and then split into the reference (50%) and sample (50%) arms. The optical length of the two arms was matched to the sample arm, and they were strictly equal to each other. A galvo scanning mirror and an achromatic lens with a focal length of 50 mm make up the scanning structure. The axial and lateral resolutions of the system in air are about 8.9 μm and 18.2 μm, respectively [37]. The detection arm consists of a spectrometer with a single line-scan camera (C-1235-1385, Wasatch Photonics, Logan, UT, USA), which contains 2048 pixels.

The BioTac sensor (SynTouch Inc., Montrose, CA, USA) is placed at the sample arm for measuring the pressure of a finger contacting a sensor, which is a commercially available fingertip-shaped sensor and sampled at a rate of 100 Hz [38]. In addition, a 3 mm thick piece of glass was placed at the fingertip to flatten it, just as a surface fingerprint scan using a traditional fingerprint scanner. To reduce noise, the glass was tilted at a small angle of 5 degrees. In order to construct the 3D image, as shown in Figure 1a, consecutively 400 cross-sectional OCT images were acquired, as shown in Figure 1b, with a beam position increment of 25 μm.

### 2.2. Key Algorithm Based on Internal Fingerprint-Guided Epidermal Thickness

**Figure 2:**
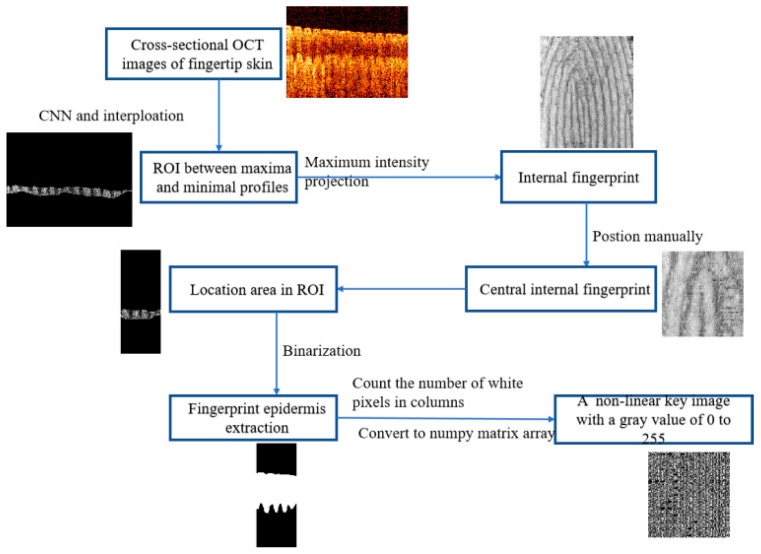
Flowchart for generating the new key.

Figure 2 demonstrates the flowchart of our new key generation process. A set of 400 OCT images of cross-sectional fingertip skin were fitted with U-Net of CNN and local interpolation to obtain the regions of interest (ROIs). These ROIs were projected with maximum intensity to obtain internal fingerprints. Then, we manually locate the central region of the internal fingerprint and select the corresponding ROIs. Finally, the epidermal fingerprints of the ROIs were binarized; the number of white pixels was counted by column, and then they were non-linearly converted into a grayscale image of 0–255. This is an optical image encryption key based on the internal fingerprint-guided epidermal thickness of fingertip skin. We describe each of the steps in detail in the following sections.

**Figure 3:**
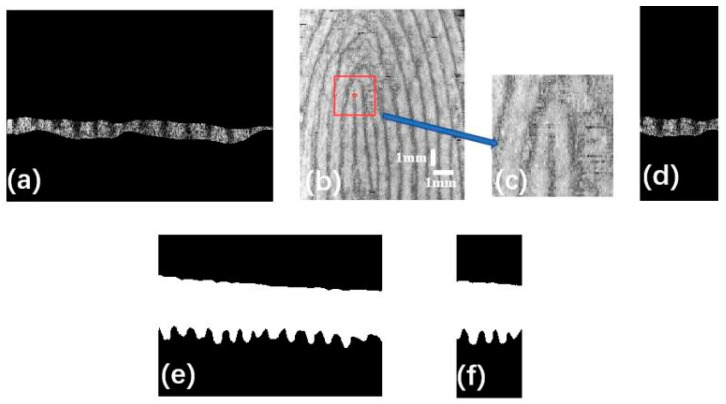
The process of epidermal fingerprint thickness extraction: (**a**) ROI of the internal fingerprint in a cross-sectional OCT image; (**b**) projection of the DEJ boundary to acquire the internal fingerprint in an en-face OCT image; (**c**) magnification of the location area of the red square in (**b**); (**d**) location area in ROI; (**e**) the upper and lower boundaries of the epidermis; and (**f**) part of (**e**).

The method of extracting internal fingerprints is similar to our previous work [35]. The boundary of the epidermis–dermis junction (DEJ) was determined and segmented by U-net [33]. Firstly, the ridge tops and papillae valleys were located by searching for the local maxima and minima. Then, the envelope curves of the local maximum and minimum values of the ridge boundary were determined by the interpolation of the ridge part of the DEJ. Finally, the region between the local maximum and minimum was selected as the ROI, as shown in Figure 3a, and the maximum intensity projection (MIP) was then applied to the ROI to extract internal fingerprints. Consecutive 400 MIP images of the DEJ boundary were used to extract internal fingerprints, as shown in Figure 3b.

The coordinates of the inner fingerprint center point were manually located by a red circle. Since there is no open-source automatic detail extraction algorithm for OCT fingerprint images, manual detail labeling was chosen in our study. In addition, the robustness of the automatic algorithm is not enough, especially for fingerprint datasets with different modalities [39].

In the internal fingerprint image, the center coordinate point was taken as the origin, as shown in the red point in Figure 3b. A square area of 101 pixels × 101 pixels is formed by taking a distance of 50 pixels from the center point along the upper, lower, left, and right edges, as shown in the red square of Figure 3b, and then the magnification of the red square is shown in Figure 3c. In our study, the MIP principle was applied to select the strongest cross-sectional signal from multiple images for superposition. The MIP algorithm makes the internal fingerprint image not affected by depth. A set of 400 3D OCT images of fingertip skin of 345 pixels × 248 pixels was processed by MIP to obtain a 345 pixels × 400 pixels cross-sectional image of the fingertip skin, which is a fingerprint image. A total of 101 images out of the 400 3D OCT images were selected to be projected on the ROI images, corresponding to the 101 images with the maximum intensity, as displayed in Figure 3d. The results can be used to verify the accuracy of the selected area.

The corresponding part of the fingertip epidermis can be determined by the region of the internal fingerprint. The processing is similar to our previous work [33]. In detail, the boundaries of the upper and lower epidermal layers in fingertip skin were determined and segmented by U-Net, as shown in Figure 3e. The 101 ROI images selected above correspond to 101 3D OCT epidermal images of fingertip skin. Similarly, part of the corresponding 101 fingertip epidermal images can be captured, as displayed in Figure 3f.

**Figure 4:**
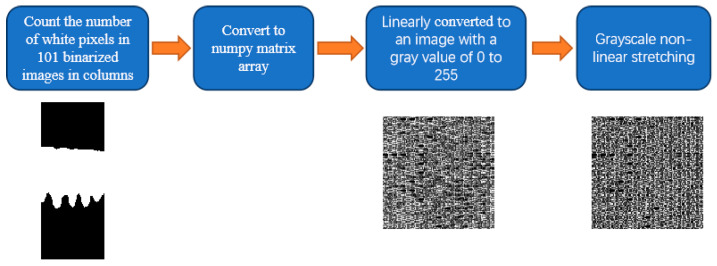
Flowchart of key generation.

After fingerprint epidermis extraction, the resolution of 101 cross-sectional fingertip epidermal images is 101 pixels × 248 pixels. And the obtained image is a binary image; that is, there are only black and white parts, and the gray values of the pixels are 0 and 255. The black area is the background with a gray value of 0, while the white area is the fingertip epidermis with a gray value of 255. Figure 4 shows the key generation method. The number of white pixels (the thickness of the fingerprint epidermis) of the 101 images in the column was counted, recorded, and saved as a 101 pixels × 101 pixels numpy matrix array. This matrix array was then converted into a linear image with a gray value of 0 to 255 and stretched non-linearly in gray scale. The resolution of the non-linear image is also 101 pixels × 101 pixels. Taking this non-linear image as a key, we defined the key as a fingerprint-guided 3D skin thickness map.

Non-linear stretching of image grayscale [40] is commonly used in digital image processing, which is defined as follows:

DB = (DA)^2^/255 (1)

where DA is the pixel value of each point in the image before the transformation, and DB is the pixel value of each point in the image after the transformation.

### 2.3. Image Encryption with MIP-Based Thickness Map

**Figure 5:**
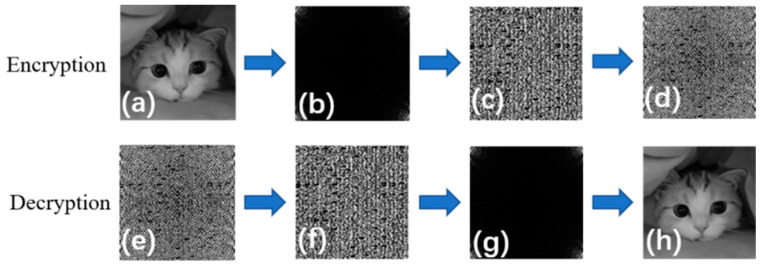
The process of encryption and decryption (**a**) image of a cat, (**b**) DCT of a cat image, (**c**) encrypted key image of MIP-based thickness, (**d**) encrypted image of a cat using fusion, (**e**) encrypted image of a cat, (**f**) decrypted key image of thickness is the same as (**e**), (**g**) DCT spectrum, and (**h**) decrypted image of a cat by inverse DCT.

The 3D fingerprint-based epidermal thickness information has been applied to implement the image encryption scheme in the intensity domain [41] and spectral domain [42], respectively. In order to further illustrate the advantage of fingertip epidermal thickness based on MIP localization for image encryption, optical encryption technology based on spectral domain image fusion was employed in our study. Its principle is to perform a discrete cosine transform (DCT) on cat ordinary images and then compress the DCT information using a low-pass filter. The detailed processes of encryption and decryption are represented in Figure 5. Figure 5a is a plain image of a cat. Figure 5b shows the DCT of images of a cat. It is proven that the image can be developed from the encryption algorithm by multiplying the DCT image of the cat image with the key image of epidermal thickness. Figure 5c displays the encrypted key image of MIP-based thickness. Figure 5d shows an encrypted image of a cat using fusion. Decryption is the opposite process of encryption. Figure 5e–h display an encrypted cat image, a decryption key image of thickness, a DCT spectrum image, and a decrypted image of cat by inverse DCT, respectively.

### 2.4. Similarity Evaluation of Images

The structural similarity index matrix (SSIM) [43] and normalized cross-correlation (NCC) were applied to evaluate the similarity between two images I and $I^{\prime }$. SSIM was defined as follows:

(2) $$SSIM\left(I,I^{\prime }\right)=\frac{\left(2\mu_{I}\mu_{I^{\prime }}+C_{1}\right)\cdot \left(2\sigma_{{II}^{\prime }}+C_{2}\right)}{\left(\mu_{I}^{2}+\mu_{I^{\prime }}^{2}+C_{1}\right)\cdot \left(\sigma_{I}^{2}+\sigma_{I^{\prime }}^{2}+C_{1}\right)}$$

where $\mu_{I}=\sum_{n=N}I\left(n\right)/N$, $\sigma_{I}^{2}=\sum_{n=N}{\left[I\left(n\right)-\mu_{I}\right]}^{2}/N$, $\mu_{I^{\prime }}=\sum_{n=N}I^{\prime }\left(n\right)/N$, $\sigma_{{II}^{\prime }}=\sum_{n=N}\left[I\left(n\right)-\mu_{I}\right]\left[I^{\prime }\left(n\right)-\mu_{I^{\prime }}\right]/N$, $\sigma_{I^{\prime }}^{2}=\sum_{n=N}{\left[I^{\prime }\left(n\right)-\mu_{I^{\prime }}\right]}^{2}/N$, C_1_ and C_2_ are two small normal numbers, N is the number of pixels in the image, I(n) and I′(n) represent the nth pixel corresponding to the two related images. And the NCC was expressed as follows:

(3) $$\mathrm{NCC}=\sum_{n=N}I\left(n\right)\cdot I^{\prime }\left(n\right)/\left[\sqrt{\sum I{\left(n\right)}^{2}}\sqrt{\sum I^{\prime }{\left(n\right)}^{2}}\right]$$

### 2.5. Random Analysis of Key

In optical encryption systems, the key is represented in the form of a random grayscale image. The pixel values in the faked image have high randomness. So, a faked key is difficult to distinguish from a real one. Three randomness parameters of image encryption, namely gray histogram, adjacent pixel correlation, and image entropy, are applied for a qualitative evaluation of the key in our study [44,45,46,47,48,49]. An image histogram is one of the important parameters for describing image features. It can intuitively display the number of pixels in each gray range of the image; that is, it can reflect the frequency of a certain gray intensity in the image. The gray histogram of the random image is uniformly distributed in the whole gray range. In an image, the degree of correlation between adjacent pixels is reflected by the correlation of adjacent pixels, which is defined [45] by the following:

(4) $$COR=\frac{\sum_{i=1}^{J}\left(x_{i}-\frac{1}{J}\sum_{i=1}^{j}x_{i}\right)\left(y_{i}-\frac{1}{J}\sum_{i=1}^{J}y_{i}\right)}{\sqrt{\sum_{i=1}^{J}{\left(x_{i}-\frac{1}{J}\sum_{i=1}^{j}x_{i}\right)}^{2}\times \sum_{i=1}^{J}{\left(y_{i}-\frac{1}{J}\sum_{i=1}^{J}y_{i}\right)}^{2}}}$$

where *J* is the number of randomly selected adjacent pixel pairs and $\left(x_{i},y_{i}\right)$ are the intensity values of adjacent pixel pairs. In general, the correlations of horizontal, vertical, and diagonal adjacent pixels of a 2D-pixelated array of images should all be analyzed. The lower the correlation value, the greater the randomness. Image entropy is a statistical form of feature that reflects the average amount of information in an image. Entropy is a measure of chaos and disorder. The higher the entropy, the greater the disorder. For a 256-level grayscale image, the entropy is calculated by the following:

(5) $$H\left(G\right)=-\sum_{i=0}^{255}P\left(G_{i}\right)\log_{2}P\left(G_{i}\right)$$

where $P\left(G_{i}\right)$ is the probability of each grayscale value appearing in the pattern. And the theoretical maximum entropy value of an 8-bit image is 8. The higher entropy of the pattern indicates the higher randomness of the pattern.

## 3. Results

### 3.1. Pressure-Insensitive Epidermal Thickness of Fingertip Skin

**Figure 6:**
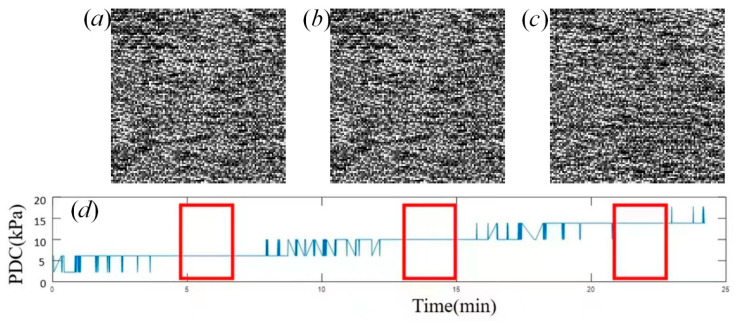
The epidermal thickness of fingertip skin at the different pressures measured by the BioTac sensor. (**a**) the epidermal thickness of fingertip skin at pressure of 6 kPa, (**b**) the epidermal thickness of fingertip skin at pressure of 10 kPa, (**c**) the epidermal thickness of fingertip skin at pressure of 14 kPa, (**d**) the three different states of pressure during sampling the fingertip skin. The three red rectangles denote the three different states of pressure during sampling the fingertip skin by OCT.

Figure 6 shows the epidermal thickness maps of fingertip skin, which were constructed using the algorithm in Figure 2 at different pressures measured by a BioTac sensor. Table 1 demonstrates that there is a large similarity between epidermal thickness maps among different pressures. Pressure status I corresponds to an approximate pressure of 6 kPa, while pressure status II corresponds to approximately 10 kPa. Pressure status III corresponds to approximately 14 kPa.

### 3.2. Application for Optical Encryption

#### 3.2.1. Feasibility Analysis

**Figure 7:**
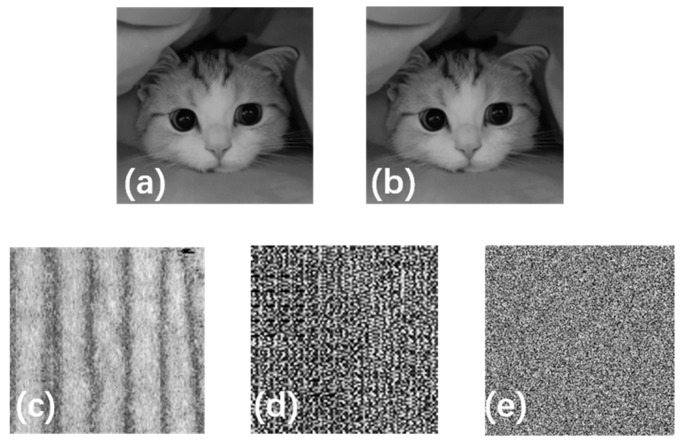
(**a**) The original image of the cat; (**b**) the decrypted image of the cat by inverse DCT; (**c**) another area of the same size in MIP; (**d**) another key image of the thickness; and (**e**) the decrypted image.

The feasibility of the image encryption method was demonstrated by numerical simulation in our study. The original image of a cat has a resolution of 256 pixels × 256 pixels. In the process of encryption and decryption, the key image with a resolution of 101 pixels × 101 pixels can be linearly transformed into an image with a resolution of 256 pixels × 256 pixels by bicuxic interpolation [50,51]. The important, relevant information of the original image can be successfully obtained through decryption when the image of internal fingerprint-guided epidermal thickness as a decryption template is completely consistent with the original key. In short, the original image can be well recovered with the correct key decryption. According to the calculation theory in Section 2.4, the SSIM value between Figure 7a and Figure 7b is 0.9670, which illustrates that the encrypted cat image information has been well extracted. That is, the image after decryption has a high similarity with that before encryption, so it can be said that the quality of the image after decryption is high.

#### 3.2.2. Security Analysis

The security of the encryption scheme was also illustrated by numerical simulation. A different part of the previous MIP location was selected to generate another 3D image of internal fingerprint-guided epidermal thickness, which was taken as the decryption template. The SSIM value between the original cat image before encryption and the after decryption image is 0.0096. The result indicates that the decryption failed with the inconsistent decryption template. Figure 7c is the key obtained from an image of the same size in a different region by MIP. The thickness information of the fingertip epidermis changes in this case. And then the epidermal thickness map generated as a decryption template (as shown in Figure 7d) is different from the previous key (as shown in Figure 5c). Therefore, the useful information of the original cat image cannot be recovered from the inconsistent encrypted image, as shown in Figure 7e. The results further illustrate that the 3D image of internal fingerprint-guided epidermal thickness can be considered important biometric information due to the different epidermal thicknesses of each person’s fingertips.

#### 3.2.3. Robustness Analysis

When fingertip skin was sampled, it suffered from the different pressures. In Section 3.1, the epidermal thickness maps were found to be insensitive to pressure. We used epidermal thickness maps at different pressures as the encrypted key and decrypted key for constructing the decrypted image, and then normalized cross-correlation between the original image and decrypted image was estimated for an analysis of image similarity for robustness. Table 2 indicates that there is high similarity between the original image and the decrypted image for the epidermal thickness map under different pressures during the encryption and decryption procedures.

**Figure 8:**
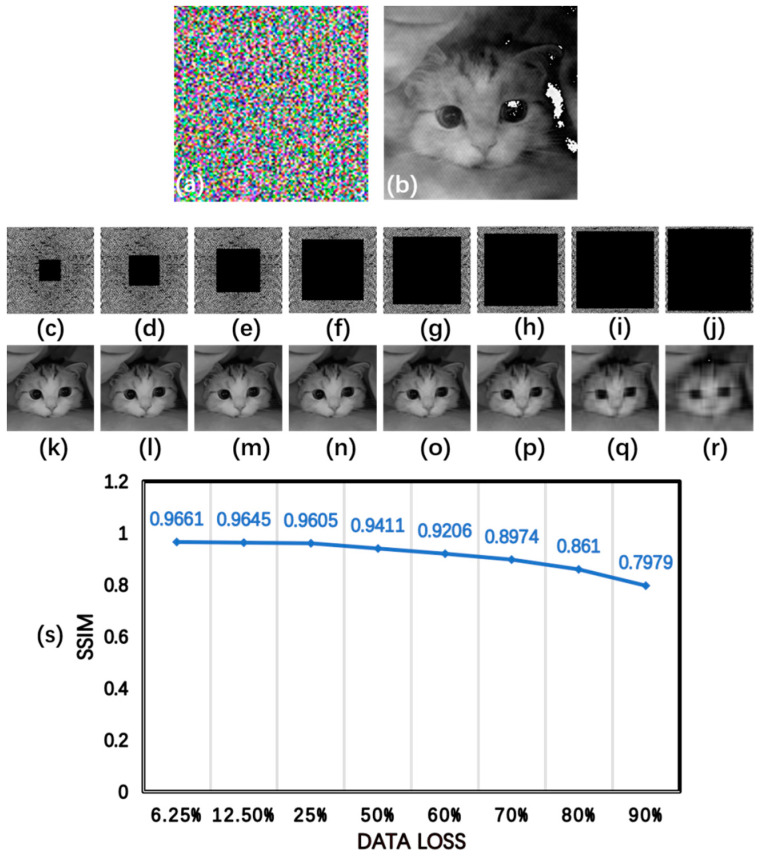
(**a**) Decryption template; (**b**) decrypted image; (**c**–**j**) encrypted images with 6.25%, 12.5%, 25%, 50%, 60%, 70%, 80%, and 90% data loss; (**k**–**r**) the corresponding decryption results of the original image; and (**s**) the relationship between the SSIM value and the data loss.

In addition, keys and encrypted images may be disturbed by noise or data, and they may be lost during the process of network transmission. So the robustness of our encryption scheme against noise attacks and data loss was tested in this study. Gaussian random noise with a mean of 0 and a variance of 0.1 was added to the key as the decryption template, as shown in Figure 8a. Figure 8b shows the cat image decrypted from the encrypted image using the consistent decryption template with noise. The SSIM value between Figure 8b and Figure 7a is 0.7121. To further investigate the robustness of our image encryption scheme against data loss, we replaced the pixel value in the middle of the encrypted image with 0. The correct key was then applied to decrypt the original image from the encrypted images with varying data loss. Figure 8c–j display the encrypted images with data loss of 6.25%, 12.5%, 25%, 50%, 60%, 70%, 80%, and 90%, respectively. And the corresponding decrypted images are shown in Figure 8k–r. The SSIM values between Figure 8k–r and Figure 7a are 0.9661, 0.9645, 0.9605, 0.9411, 0.9206, 0.8974, 0.8610, and 0.7979, respectively, as shown in Figure 8s. The results demonstrate that the decrypted image becomes blurry with an increase in the data loss. These results clearly show that our proposed image encryption method is robust against noise attacks and data loss. Important information can be recovered from the original image despite data loss.

#### 3.2.4. Key Randomness Analysis

The randomness of the key is a factor that affects the strength of the key. In a forgery attack, the randomness of the faked key directly affects the concealment of the deception action [45]. Grayscale histogram, adjacent pixel correlation, and image entropy were employed to evaluate the imperceptibility of faked keys. In our analysis, the key image of MIP-based epidermal thickness (shown in Figure 5c) generated from the fingerprint image (shown in Figure 3c) was taken as the original key, and the key image (shown in Figure 7d) generated from the fingerprint image (shown in Figure 7c) was considered the fake key.

**Figure 9:**
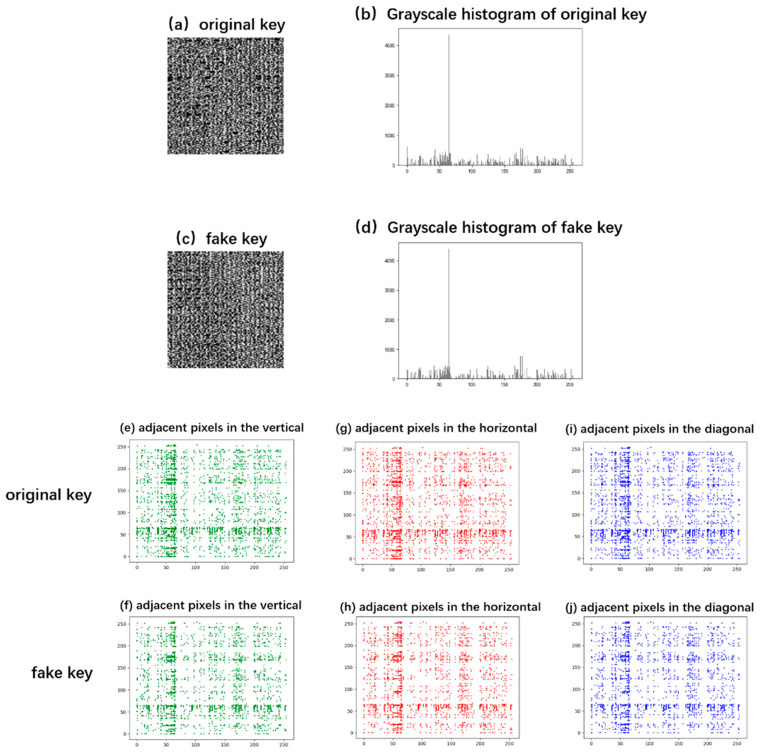
(**a**–**d**) Grayscale histograms of the original key and the fake key; intensity distribution diagrams of adjacent pixels in the vertical (**e**,**f**), horizontal (**g**,**h**), and diagonal (**i**,**j**) directions of the original key and the fake key.

Figure 9a–d displays the grayscale histograms of the original key and the fake key. From the figure, it has a similar statistical appearance between the gray histograms of the original key and the fake key. The pixel values of both the original key and the fake key are roughly evenly distributed between 0 and 255, except for 65. The result shows that the grayscale histograms are not completely uniform, which may be affected by human biological characteristics. Figure 9e–j shows the intensity distribution diagrams of adjacent pixels in the vertical, horizontal, and diagonal directions of the original key and the fake key. From the figure, the intensity distribution of the fake key and the original key is similar in three directions. The average correlation coefficients of adjacent pixelated values in three directions and the average entropy in the original key and fake key were extracted and displayed on Table 3. The absolute average correlation coefficients of adjacent pixelated values in horizontal, vertical, and diagonal directions are close to zero in both keys. From Table 3, it can also be observed that the average entropy of the image is 7.8201 in the fake key, while it is 7.8177 in the original key. These values are so close that they are indistinguishable between original and fake keys. The results demonstrate that the key generated by our proposed algorithm is very close to the random grayscale image without any information about the plaintext image. These keys are not only visually random, but they are also random quantitatively in statistics. Although a gray histogram is a little less than random digital encryption, the use of human biometric keys greatly improves the security of information encryption. The thickness of the epidermis as the key increases the complexity of the encryption structure and provides a reference for the new optical image encryption technology.

## 4. Discussion

Keys based on fingerprint images have been widely studied in optical image encryption schemes [8,34,35,36,52,53,54,55]. In this paper, we proposed a new key based on internal fingerprint-guided epidermal thickness for an encryption scheme. Compared to existing technologies, such as facial recognition, fingerprints, and iris recognition, the epidermal thickness map used in optical image encryption provides higher confidentiality due to binge informed and giving consent for obtaining the data by OCT, and it cannot be collected non-invasively without permission in daily life, meaning the privacy concerns related to the use of internal biometrics for encryption. Furthermore, some recent technological developments, such as metastructures [56], nanoparticles [57], near-zero-index materials [58], grapheme [59], and plasmonics [60], have also been applied for encryption. However, compared to the proposed OCT-based internal fingerprint-guided epidermal thickness map, the procedure for generating these structures or nanoparticles is complex and highly environmentally demanding.

Although the effects of changes in the inner layer due to external environmental factors, such as temperature, have not been discussed previously, it is worth noting that muscles typically have a coefficient of thermal expansion of 8.9 ± 1.7 × 10^−4^/°C [61]. This implies that a 1 °C increase in temperature corresponds to an approximately 0.1% increase in muscle volume. The temperature range for fingertips is not significantly large, usually around 25 ± 10 °C. As a result, the cumulative temperature-induced change in skin thickness is approximately ±1%, which is much smaller than the error discussed in the system’s robustness analysis.

The proposed encryption method, based on our new key, is applied to encrypt images. These encrypted images can be transmitted through existing digital security frameworks and protocols, achieving applications including the integration of digital certificates to verify the authenticity of encrypted images, the application of digital signatures to verify the source identity of encrypted images, and the implementation of a secure key management system to ensure the confidentiality of generated keys. Thus, the proposed method enhances the security and trustworthiness of encrypted images, allowing them to be transferred within existing digital security frameworks and protocols.

Additionally, the entire encryption and decryption process of the system can be completed within 10 s. The key image acquisition time by the OCT system is approximately 4 s, while image segmentation using U-Net takes around 4 s. Other computational times are negligible, and the system can generally meet real-time encryption/decryption requirements. Furthermore, with technological advancements, these times can be further reduced.

## 5. Conclusions

In summary, a new key based on the internal fingerprint-guided epidermal thickness of fingertip skin using OCT was proposed for optical encryption. There are three steps to the key generation algorithm. Firstly, U-Net was utilized to segment the upper and lower boundaries of the epidermal layer in cross-sectional OCT images of the fingertip skin. Secondly, the MIP algorithm was applied to extract the internal fingerprint at the DEJ of fingertip skin. Finally, the internal fingerprint-guided epidermal thickness was calculated and converted into a thickness map.

The experimental results demonstrate that the internal fingerprint-guided key based on OCT could be encrypted as important biometric information through feasibility, security, robustness analysis, and key randomness analysis. This key has the capability to address the security concerns arising from the non-invasive collection of biometric data, such as faces and fingerprints, without proper permission in daily life.

Therefore, the key formed by the internal fingerprint-guided epidermal thickness has a potential for information encryption, which can be transferred through existing digital security frameworks and protocols, such as transport layer security (TLS) and secure sockets layer (SSL). However, the potential barriers to the widespread adoption of this OCT-based biometric encryption technique are its high cost and the large volume of OCT data acquisition systems. Thus, the future research areas are cost optimization and miniaturization of OCT systems.

## Figures and Tables

**Table 1:** Normal correlation coefficients of epidermal thickness maps at the different pressures.

Different Pressures	Status I	Status II	Status III
Status I	1	0.8823	0.800
Status II	0.8823	1	0.955
Status III	0.800	0.955	1

**Table 2:** Normal correlation coefficients between the original image and the decrypted image.

	Decryption Map		
Encryption Map	Status I	Status II	Status III
Status I	1	0.9062	0.9048
Status II	0.9098	1	0.9067
Status III	0.9098	0.9081	1

**Table 3:** Average correlation coefficients of adjacent pixelated values in different directions and average entropy in the original key and the ones in the fake key.

	Correlation Coefficients			Entropy
	Horizontal	Vertical	Diagonal	
Original key	−0.0075	−0.0354	−0.0479	7.8177
Fake key	−0.0058	−0.0163	−0.0664	7.8201

## Data Availability

The data presented in this study are available on request from the corresponding author.
